# Cross-Professional Evaluation of 3D Visualization of Liver Malignancies in the Decade of AI and Automatic Segmentation: A Benefit for Multidisciplinary Teams and Tumor Board Decisions?

**DOI:** 10.7759/cureus.72320

**Published:** 2024-10-24

**Authors:** Christian Heiliger, Dorian Andrade, Lucas Etzel, Daniel Roessler, Vanessa F Schmidt, Florian Boesch, Jens Ricke, Jens Werner, Konrad Karcz, Olga Solyanik

**Affiliations:** 1 Department of General, Visceral and Transplant Surgery, University Hospital, Ludwig Maximilian University (LMU) Munich, Munich, DEU; 2 Department of Radiation Oncology, Klinikum rechts der Isar, Technical University of Munich, Munich, DEU; 3 Department of Internal Medicine II – Gastroenterology, University Hospital, Ludwig Maximilian University (LMU) Munich, Munich, DEU; 4 Department of Radiology, University Hospital, Ludwig Maximilian University (LMU) Munich, Munich, DEU; 5 Department of General, Visceral, and Pediatric Surgery, University Medical Center Göttingen (UMG), Göttingen, DEU

**Keywords:** 3d reconstruction, automatic segmentation, interdisciplinary decision making, liver malignancies, liver surgery

## Abstract

Purpose

To investigate whether automatic 3D visualization of computed tomography (CT) data sets with singular liver tumor compared to 2D images could foster a broader understanding of tumor localization and resectability in the liver within a multidisciplinary team and might therefore be a useful tool in multidisciplinary decision-making.

Material and methods

The study was configured as a web-based questionnaire. Physicians of all levels of medical training from surgery, radiology, and gastroenterology departments were recruited. A total of seven cases with singular liver tumor CT images with adequate quality were selected. Automatic 3D segmentation was performed using Universal Atlas (Release 5.0) as part of the Brainlab of Elements software suite (Brainlab AG, Munich, Germany). All cases were randomly presented in a 2D and 3D manner. After every case-presentation, multiple choice (single answer) questions concerning tumor extent and resectability were asked. The questions as well as the answers defined to be correct, were evaluated by two senior consultants from the radiology and surgery department. The primary outcome parameters were the correctness of answers stratified for medical specialty and for the level of medical training. The secondary outcome was the time needed for the evaluation of seven liver cases using 2D versus 3D images. Six additional questions were tailored to evaluate the subjective value of the 3D visualization.

Results

A total of 92 participants participated in the study, 31.5% of them were abdominal surgeons, 34.8% gastroenterologists, and 33.7% radiologists. Based on the level of medical training, 66 were residents (71.7%) and 26 consultants (28.3%). Only radiologists answered more questions correctly using 2D imaging compared to the 3D method (p = 0.006). There was no statistically significant difference between correctly answered questions when using 2D vs. 3D visualization in the gastroenterologist and surgeon groups (p > 0.05). The resident subgroup showed no statistically significant difference when using the 2D vs. 3D images (p > 0.05), the consultant subgroup answered more questions correctly using 2D imaging (p = 0.009). Physicians with elementary experience of liver pathology also showed no difference in 2D vs. 3D (p = 0.332), physicians with proficient experience of liver pathology answered more questions correctly using 2D imaging (p = 0.010). The median time taken for the evaluation of the seven liver cases was only significantly faster for the gastroenterologist group (p = 0.006) using the 3D analysis (median: 9.1 minutes) than the 2D analysis (median: 10.7 minutes).

Over 80% of the participants found the 3D presentation to be a helpful additional tool for the clinical routine according to the subjective questionnaire.

Conclusion

In this study 3D visualization of liver tumors was evaluated as helpful within a multidisciplinary team of radiologists, surgeons, and gastroenterologists. However, significantly superior results in the understanding of liver anatomy could not be demonstrated by means of 3D visualization. It may be that more immersive technologies such as augmented reality or virtual reality will lead to a superior understanding compared to conventional presentation of information in 2D cross-sectional images.

## Introduction

The increasingly differentiated understanding of tumor biology leads to more multimodal and multidisciplinary-oriented therapy of liver malignancies. This requires close coordination among medical disciplines to offer an optimal and highly individualized treatment for the patient. Especially patients with initially non-resectable disease may benefit from multidisciplinary approaches, as complementary systemic and/or additional local ablative therapeutic modalities may enable curative-intended surgery [1-3].

In the case of local ablative pretreatment and/or surgical methods, an accurate understanding of complex liver anatomy plays a crucial role. A fundamental aspect of surgical planning is the identification of vascular tributaries and detailed morphological features of liver lesions, which can be reflected in computed tomography (CT)-based 3D models [4-7].

Even though there are international consensus recommendations suggesting the application of preoperative 3D visualization for diagnosis and management of liver diseases [8], there are no clear standards that define the accuracy of 3D models, the type of segmentation, and their clinical reliability [9]. 3D reconstructions in the clinical setting are still performed using manual or semi-automated techniques, which require a significant amount of time for segmentation. Nevertheless, by applying novel methods of machine learning and artificial intelligence, it could be possible to display 3D objects with sufficient accuracy from CT and MRI by automatic segmentation, without the need for manual segmentation [10-15].

Understanding the difficult liver anatomy within multidisciplinary tumor boards requires appropriate experience of medical colleagues. However, an understanding of the different therapeutic options, including possible surgical resections, ablative procedures, and other treatments such as transarterial chemoembolization (TACE), selective internal radiation therapy (SIRT), and portal vein embolization (PVE), as well as their contraindications due to anatomical conditions, is usually limited to a small part of the interdisciplinary team. Particularly younger colleagues are excluded from the discussion.

The aim of this study was to compare the accuracy of a 3D reconstruction of CT data sets with singular liver tumors based on an advanced (semi-)automated post-processing software versus 2D images for the understanding of liver tumor localization, depending on the medical specialty and level of training. We further investigated whether the 3D method leads to a better understanding of pathology, and whether it may serve as a useful tool for multidisciplinary team decision-making.

## Materials and methods

Study design and participants

The study was configured as a web-based questionnaire. This study has been approved by the ethics committee at the Ludwig Maximilians University (EK-LMU19-395). The free online survey application (LimeSurvey, formerly PHPSurveyor) was used for the study. Residents, consultants and senior consultants from visceral surgery, radiology and internal medicine departments at a single-center university hospital were recruited for participation in the study. The study was conducted between July 2019 and April 2020. The participants performed the study on a standard personal computer without permission to use other content from the internet or other sources of information. An investigator was present during the study survey to support the technical part of the questionnaire.

Study setting and protocol

The inclusion criteria were as follows: patients who underwent traditional triphasic CT scans with adequate image quality and without artifacts, had no history of prior hepatic treatment, and had a singular liver tumor. A total of seven cases with singular liver tumors showing different tumor localizations were selected for the study. All image data were acquired with a CT scanner (Somatom Force, Siemens AG, Erlangen, Germany) at the radiological department of our institution. The liver scans were performed in arterial, portal, and venous phase using bolus tracking contrast agent application (1.5 mL/kg of a nonionic iodine-containing contrast agent, Ultravist 370, Schering AG, Berlin, Germany; flow rate 3-4 mL/s). CT examinations were performed using the following parameters: tube voltage, 120 kVp; detector collimation, 0.625 to 1.250 mm; table pitch, 1:0.984 to 1.375; matrix, 512 x 512; and reconstruction intervals, 3.00 mm. 

Automatic contouring of the liver was performed by Universal Atlas (Release 5.0) as part of the Brainlab Elements software suite (Brainlab AG, Munich, Germany). 

This segmentation algorithm is based on a Synthetic Tissue Model (Atlas), i.e. a universal atlas with tissue-specific meta-information for each voxel, such as tissue class and related elasticity and density. Nonlinear, elastic image fusion is used for registration of the Synthetic Tissue Model with the patient scan. Body parts and specific anatomical features are detected. In a second step, Modality Simulation is achieved by converting the anatomically adapted model into a patient and tissue-specific gray-scale value simulation of the evaluated scan. Due to the flexibility of the model, multiple CT scans (or MRIs), acquired with the same frame of reference may be exploited simultaneously in terms of image information. The scans are iteratively registered to the set of modality-specific atlases (of the Synthetic Tissue Model). During this process, the matching of individual structures or regions is accomplished by individual weighting of each atlas-to-scan registration, depending on the corresponding scan properties. After registration of the adapted model to one or more datasets, post-processing methods such as shape-preserving smoothing and image intensity-based registration optimization are applied to improve the accuracy of the segmentation.

**Figure 1:**
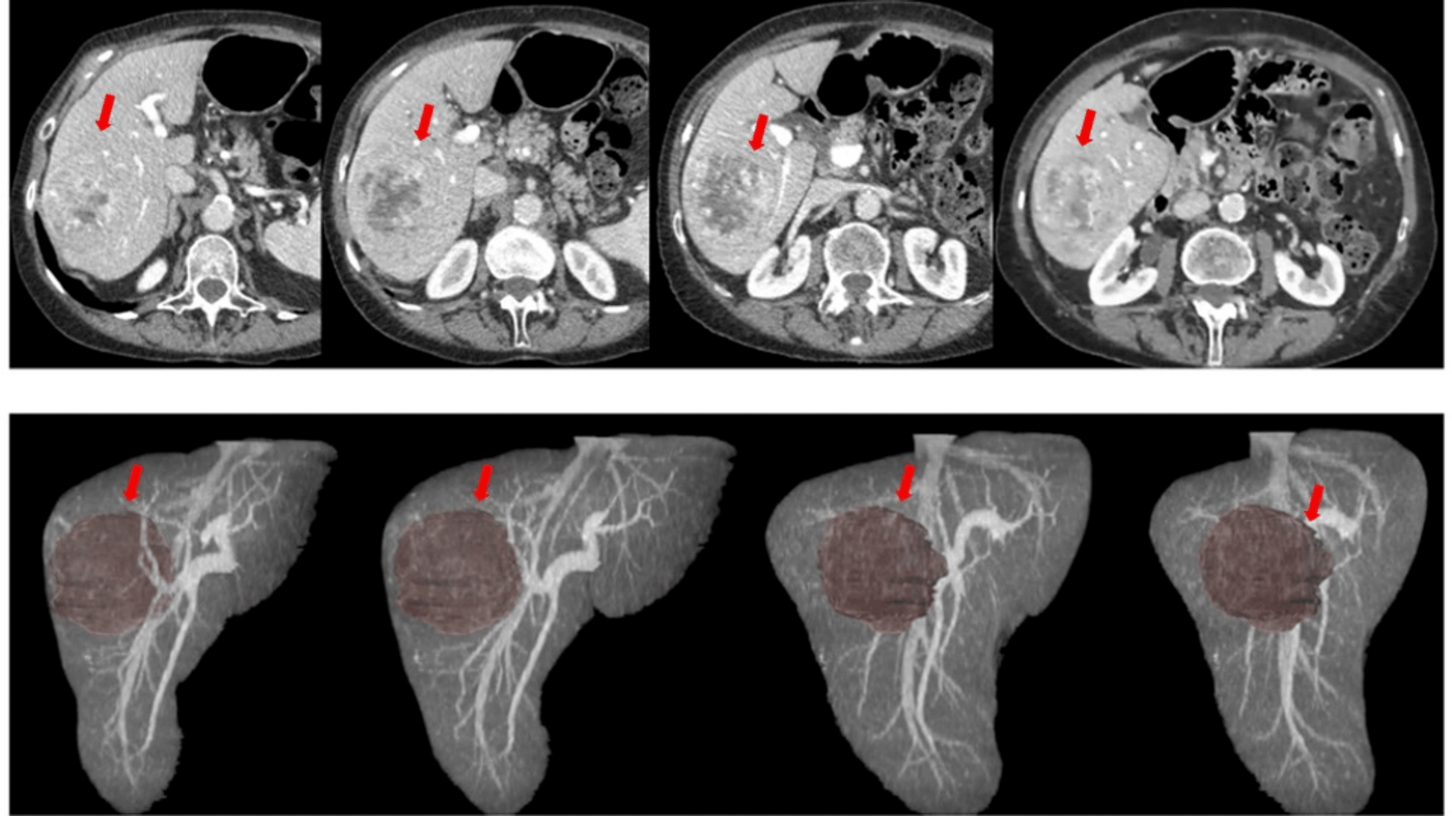
2D and 3D visualization of the liver 76-year-old male patient with a singular liver tumor in the right liver lobe. 2D axial abdominal CT images (top row) and corresponding semiautomated 3D reconstructions of the same patient (bottom row).

For each of the seven imaging cases, one multiple-choice (single answer) question was created concerning the assessment of tumor extent or tumor resectability, in order to simulate the preoperative planning and decision-making process during a multidisciplinary tumor conference (Figure 1). The questions posed, as well as the answers defined to be correct, were evaluated by two senior consultants from the radiology and surgery department with over 10 years of individual experience for liver pathologies. Each question was posed twice in the online questionnaire survey, once each for 2D and 3D images. The questions appeared in a random numerical sequence, thus avoiding back-to-back occurrence of the same question. Both the binary results (correct/not correct) as well as the time needed to answer a respective question with 2D or 3D images were recorded automatically. At the beginning of the online survey, all participants were asked to specify their specialty, their current year of experience in the specialty and to conduct the self-assessment regarding their experience in liver pathology. The primary outcome parameters were the accuracy of answers depending on the medical specialty, on the level of medical training and on the self-assessment regarding the experience of liver pathology. Time taken for the evaluation of seven liver cases using 2D versus 3D images was the secondary outcome. 

At the end of the online survey participants were asked to complete six evaluative questions assessing the subjective value of 3D visualization and its utility concerning the comprehension of the anatomy and the surgical steps compared to 2D visualization (Appendix). 

Data analysis 

Data transformation and analysis as well as graph creation was conducted dynamically and reproducibly using the R software environment for statistical computing and graphics (version 4.0.0) with R Markdown and selected R packages (R Foundation for Statistical Computing, Vienna, Austria). 

To assess the utility of 2D and 3D imaging for the comprehension of the tumor's localization and extent, the results of correct answers to questions with liver pathology (n = 7 using 2D imaging, n = 7 using 3D imaging) were represented as a percentage value. Thereafter, the data was sorted into groups based on the specialty of participants and their level of training. Sample normality was analyzed using q-q plots and the Shapiro-Wilk test. The Wilcoxon signed rank test with continuity correction was used to compare paired continuous data samples, while calculating the estimated differences between the sample medians with 95% confidence intervals. Boxplots were used to visualize the distribution of continuous data. A p-value < 0.05 was considered statistically significant.

Answers to five evaluative questions were presented using a 5-point Rating scale (Likert scale) with regards to how the 3D imaging was helpful in the comprehension of the tumor extent and its resectability compared to 2D visualization (1 point = none; 5 points = very helpful). The sixth evaluative question was addressed to assess which imaging method would be preferred by participants to quickly understand the relationship of the liver tumor to the liver veins (2D; 3D; 2D and 3D).

## Results

Study participants

A total of 92 physicians, of which 29 (31.5%) were surgeons, 32 (34.8%) were gastroenterologists and 31 (33.7%) were radiologists, participated in the study. The participant population is listed in Table 1. 

**Table 1:** Primary outcome parameters. Evaluation of correct answers of seven liver cases using the 2D- and 3D-based analysis Wilcoxon signed rank test with continuity correction was used to compare paired continuous data samples, while calculating the estimated differences between the sample medians with 95% confidence intervals. A p-value < 0.05 was considered statistically significant.

Participants	n (%)	2D (median score/ IQR) %	3D (median score/ IQR)	p	Estimated difference in location of the medians / 95% CI (%)
All	92	85.7 / 14.3	71.4 /28.6	p = 0.001	14.3 / 0 to 21.4
Subgroup analysis based on medical speciality					
Surgeons	29 (31.5)	85.7 / 14.3	85.7 / 14.3	p = 0.854	0 / -14.3 to 14.3
Gastroenterologists	32 (34.8)	71.4 / 17.9	71.4 / 17.9	p = 0.353	14.3 / 0 to 21.4
Radiologists	31 (33.7)	85.7 / 35.7	57.1 / 14.3	p = 0.006	21.4 / 7.1 to 28.6
Subgroup analysis based on the level of medical training					
Residents	66 (71.7)	71.4 / 14.3	71.4 / 28.6	p = 0.081	14.3 / 0 to 21.4
Consultants	26 (28.3)	85.7 / 14.3	71.4/28.6	p = 0.009	14.3 / 0 to 28.6
Subgroup analysis based on the experience of liver pathology					
Elementary	70 (76.1)	71.4 / 28.6	71.4 / 28.6	p = 0.332	7.1 / 0 to 14.3
Proficient	22 (23.9)	85.7 / 10.7	71.4 / 28.6	p = 0.010	21.4 / 7.1 to 35.7

Results of correct evaluation of seven liver cases using the 2D- and 3D-based analysis

**Figure 2:**
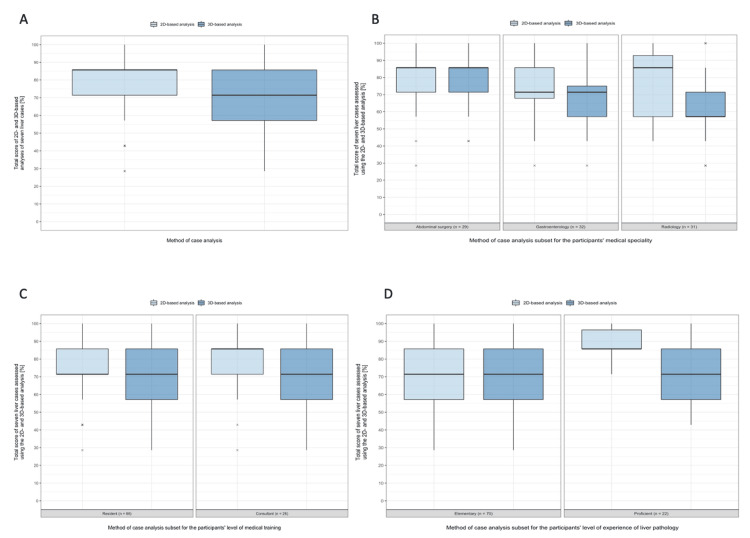
Primary outcome parameters: Boxplots depicting results of correct evaluation of seven liver cases using the 2D- and 3D-based analysis A. Evaluation of seven liver cases assessed using the 2D- and 3D-based analysis for all participants. B. Evaluation of seven liver cases assessed using the 2D- and 3D-based analysis dependent on the participants’ medical specialty. C. Evaluation of seven liver cases assessed using the 2D- and 3D-based analysis dependent on the participants’ level of medical training. D. Evaluation of seven liver cases assessed using the 2D- and 3D-based analysis subset for the participants’ level of experience of liver pathology. Wilcoxon signed rank test with continuity correction was used to compare paired continuous data samples, while calculating the estimated differences between the sample medians with 95% confidence intervals. A p-value < 0.05 was considered statistically significant.

Overall, the participants answered more questions correctly when using the 2D imaging vs. 3D imaging. This difference was statistically significant (p = 0.001). The radiologists answered more questions correctly using 2D imaging compared to the 3D method (p = 0.006). There was however no statistically significant difference between correctly answered questions using 2D versus 3D imaging by gastroenterologists (p = 0.353) and by surgeons (p = 0.854) (Table 1, Figure 2).

Comparison of subgroup analysis based on level of medical training

For the resident subgroup, there was no statistically significant difference (p = 0.081) between correctly answered questions using 2D and 3D imaging.

The consultant subgroup answered more questions correctly using 2D imaging compared to 3D method (p = 0.009). 

Comparison of subgroup analysis based on experience of liver pathology

There was no statistically significant difference between correctly answered questions when using 2D and 3D imaging by physicians with elementary experience of liver pathology (p = 0.332). The physicians with proficient experience of liver pathology answered more questions correctly when using 2D imaging than with the 3D reconstructions (p = 0.010) (Figure 2).

Results of time taken for the evaluation of seven liver cases assessed using the 2D and 3D imaging

**Figure 3:**
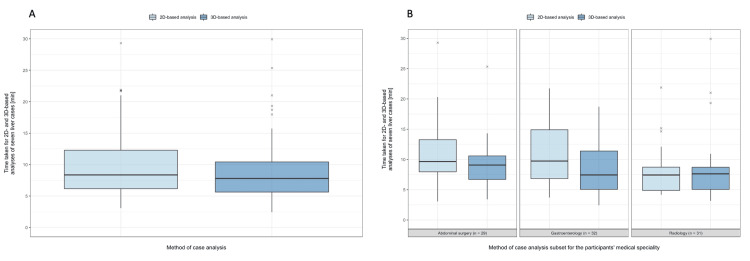
Secondary outcome parameters: Boxplots depicting the time taken for the evaluation of seven liver cases assessed using the 2D- and 3D-imaging A. Time taken for the evaluation of seven liver cases assessed using the 2D- and 3D-based analysis for all participants. B. Taken for the evaluation of seven liver cases assessed using the 2D- and 3D-based analysis subset for the participants’ medical specialty. A total of 11 values (6.0%) above 30 minutes were excluded from graphical representation. Wilcoxon signed rank test with continuity correction was used to compare paired continuous data samples, while calculating the estimated differences between the sample medians with 95% confidence intervals. A p-value < 0.05 was considered statistically significant.

The median time taken for the evaluation of the seven liver cases was not significant in the radiologist (p = 0.854; median 2D 7.6 minutes vs. median 3D 7.6 minutes) and surgeon (p = 0.143; median 2D 9.7 minutes vs. 3D 9.4 minutes) group. The gastroenterologist group (p = 0.006) needed more time to answer the questions using the 2D analysis (median: 10.7 minutes) than with the 3D analysis (median: 9.1 minutes) (Table 2, Figure 3).

**Table 2:** Secondary outcome parameters. Evaluation of time taken for the answer of seven liver questions using the 2D- and 3D-imaging Wilcoxon signed rank test with continuity correction was used to compare paired continuous data samples, while calculating the estimated differences between the sample medians with 95% confidence intervals. A p-value < 0.05 was considered statistically significant.

Participants	2D (median score/ IQR) minutes	3D (median score/ IQR) minutes	p	Estimated difference in location of the medians/95% CI (minutes)
All	8.9 / 8.4	8.0 / 5.3	p = 0.010	0.9 / 0.2 to 1.8
Surgeons	9.7 / 6.8	9.4 / 4.2	p = 0.143	0.9 / 0.2 to 1.8
Gastroenterologists	10.7 / 8.7	9.1 / 7.3	p = 0.006	2.2 / 0.7 to 5
Radiologists	7.6 / 4.0	7.6 / 3.7	p = 0.854	-0.1 / -0.9 to 0.7

Subject evaluation

**Figure 4:**
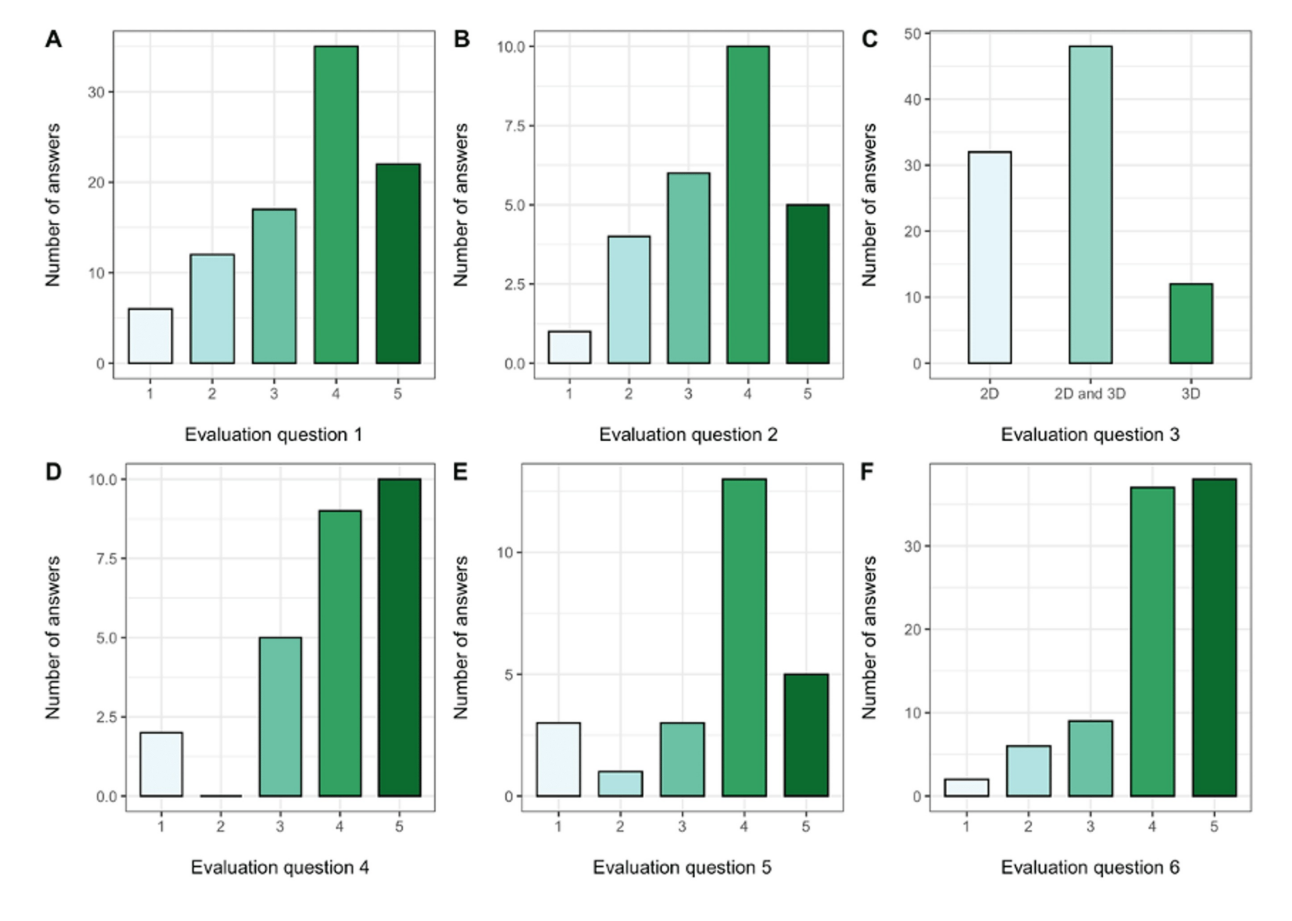
Evaluation questions. Bar charts depicting the respective number of answers given for each of the six evaluation questions. Unless otherwise stated, 1 =none and 5=very helpful. A. How helpful was the 3D reconstruction in understanding the anatomy? Please rate the utility of the 3D view? (57 (61.96%) participants responded helpful or very helpful) B. How helpful was the 3D reconstruction in defining a possible resection border? 15 (57.69%) surgeons rated the 3D visualization as helpful or very helpful in evaluating of resection margins (answered only by surgeons). C. To quickly visualize the pathology in relation to the vessels, would you preferentially use: 2D, 2D and 3D, or only 3D? 48 (52.17%) study participants would prefer a combination of 2D and 3D D. Do you think that a 3D reconstruction on an intraoperative monitor would help to identify the resection border? 19 (73.07%) surgeons would rate a 3D reconstruction on an intraoperative monitor as helpful or very helpful (answered only by surgeons) E. Do you think that a 3D reconstruction on an intraoperative monitor would help to identify the correct approach for biopsy / ablation? 18 (72%) of the participating radiologists would rate 3D visualization helpful or very helpful for biopsies or ablation (answered only by radiologists) F. Do you think that a 3D reconstruction in addition to the 2D data set, would help to understand better the anatomy during tumorboard meetings? 75 (81.52%) participants responded as helpful or very helpful.

According to the results of the evaluative questions, 3D visualization was assessed to be regarded as a useful tool for the comprehension of hepatic anatomy and planning of further surgical steps. Furthermore, it should be used in addition to standard 2D imaging (Figure 4).

## Discussion

Nowadays, there are various studies available supporting the beneficial role of 3D imaging for liver anatomy and liver surgery [5,7,16,17]. However, in the clinical routine and within the multidisciplinary tumor conferences, radiological images are most often presented in 2D. The first goal of this study was to examine whether the 3D imaging with singular liver tumor compared to 2D images is more accurate and more helpful to understand the liver tumor's localization for physicians with different medical specialties, different levels of medical training and different experience in liver pathology. The second goal of this study was to determine whether seven liver cases using 3D images could be constructed faster than those with 2D images by physicians. 

In the study setup conducted with 92 physicians, the following results were observed. Firstly, the radiologists solved more cases correctly using 2D imaging, whereas gastroenterologists and surgeons answered questions equally correctly using 2D and 3D imaging. Secondly, both the consultants and the physicians with proficient experience in liver pathology answered more questions correctly using 2D imaging as with the 3D reconstructions. For residents and participants with elementary experience in the liver pathology were 3D and 2D representation equivalent. Thirdly, although the 3D presentation showed no better results than 2D visualization, over 80% of the participants found the 3D presentation as a helpful additional tool for the clinical routine according to the subjective questionnaire. Moreover, in the operating room or during interventions, the 3D presentation of the liver pathology could be an advantage according to 70% of the surgical and radiological participants. Most of the participating surgeons found 3D visualization helpful to identify resection margins and would advocate 3D visualization in the operating room, also representing the sub-cohort relatively scoring best in 3D imaging.

Hence, one of the key findings of this study is well known in medicine generally: It emphasizes the relevance of experience for both, the pathologies one’s sub-specialization is confronted with and its diagnostic means. This is substantiated by the fact that participants working with 2D imaging of the liver regularly on a high-frequent basis scored connotatively better and faster than those with less experience. As none of the participants worked with hepatic 3D imaging regularly before, the beneficial impact of 3D imaging within our study can only be hypothesized. Yet, one could argue that the similar results between 2D and 3D imaging in the less 2D-experienced participant cohort at least proved a non-inferiority of the 3D technique. The comparison between 2D axial CT images and the semiautomated 3D reconstructions in Figure 1 effectively highlights the potential advantage of 3D visualization in appreciating the spatial relationships and precise localization of the liver tumour. However, the top-down 2D view remains essential for detailed anatomical analysis, indicating that the combined use of both 2D and 3D imaging could enhance preoperative planning by leveraging the strengths of each modality.

However, when taking into account the coherence of clinical benefit of 3D imaging and its importance to patient outcomes, the data in the literature is sometimes contradictory. As such, Mise et al. demonstrated in 1194 living donor liver transplantation that using 3D virtual surgical planning software preoperatively leads to better outcomes than without [20]. At the same time, Takamoto et al. showed no significant advantage of using 3D planning preoperatively with 473 patients [9].

In various prior articles manual segmentation was used [5,16-19]. For the 3D reconstruction in our work, we used an automatic approach, which may allow a seamless integration into a clinical routine without time expenditure for the manual segmentation. In addition to that, the liver questions with 3D imaging were answered faster than those with 2D images by all participants, although in part without significant differences. So, the integration of 3D imaging using an advanced (semi-) automatic segmentation protocol in the daily routine can lead to faster interpretation of information as even less experienced physicians might find it easier to envision certain anatomic occurrences. Finally, most of the participants find a 3D representation helpful.

Our study had some limitations. The most significant limitation of this work is that it is a single center study with a small number of participants. Secondly, in the data presented here, we have not actually used the “real” three-dimensional visualization. Instead, we examine, as some other studies, a “pseudo” 3D effect, which is based on shadows and different grades of transparency. The “real” 3D images could, i.e., be achieved while looking through Virtual Reality 3D glasses. Furthermore, the accuracy of the segmentation of the software and the influence of the choice of potentially different software were not analyzed. Furthermore, only patients with individual liver lesions were included in the study; in particular, more complex metastasis patterns, e.g. in colorectal carcinoma, are of high clinical relevance and should be further investigated. Moreover, the questions for seven liver cases were presented either with 2D imaging or 3D imaging, there were no questions with both imaging methods concurrently. Furthermore, the number of anatomical examples examined is quite small with only seven different cases. Due to the practicability of the implementation and a maximum number of study participants, a larger number of anatomies to be examined was not used here.

## Conclusions

In this study, 3D visualization of liver tumors is evaluated by a multidisciplinary team of radiologists, surgeons and internists as a helpful tool. A faster acquisition of anatomy and a common understanding of all involved, even the less experienced, seems to be improved with the help of 3D visualization.

However, significantly superior results in the understanding of liver anatomy could not be demonstrated by means of 3D visualization in this study. Therefore, we conclude that more immersive technologies such as augmented reality will lead to a superior understanding compared to conventional presentation of information in 2D cross-sectional images. Moreover, further prospective studies analyzing an immersive technology and its influence of clinical outcome parameters should be investigated, especially within a multidisciplinary setting.

## Appendices

Full questionnaire:

http://dx.doi.org/10.6084/m9.figshare.27174237
